# Diagnosis and treatment of calciphylaxis in patients with chronic kidney disease

**DOI:** 10.1590/2175-8239-JBN-2021-S111

**Published:** 2021-12-03

**Authors:** Leandro Junior Lucca, Rosa Maria Affonso Moysés, Adriano Souza Lima

**Affiliations:** 1 Universidade de São Paulo, Hospital de Clínicas da Faculdade de Medicina de Ribeirão Preto, Ribeirão Preto, SP, Brazil.; 2 Universidade de São Paulo, Faculdade de Medicina, Hospital das Clínicas, Pathophysiology Laboratory, São Paulo, SP, Brazil.

1. Calciphylaxis is a rare but very serious disease associated with high morbidity and mortality. It most commonly affects patients undergoing dialysis, but it may occur in individuals with chronic kidney disease (CKD) under conservative treatment, transplant recipients, and even in those without kidney disease (Evidence).

2. Its pathophysiology is not completely known. However, histological findings show calcification of arterioles, besides thrombosis and endothelial damage of these vessels. These changes cause ischemia and necrosis of the subcutaneous tissue, with necrotic ulcers appearing in more advanced stages (Evidence).

3. The diagnosis of calciphylaxis is clinical. Calciphylaxis should be suspected in CKD patients presenting with nodular, purpuric/erythematous lesions or painful subcutaneous plaques, livedo reticularis, non-healing ulcers and/or skin necrosis, especially on the thighs and other areas of increased adiposity (Evidence). 

4. Risk factors associated with the onset of calciphylaxis are: female gender, diabetes *mellitus*, warfarin use, obesity, hypoalbuminemia, and alterations in mineral metabolism [hypercalcemia, hyperphosphatemia, and parathyroid hormone (PTH) extremes] (Evidence).

5. Skin biopsy should be performed in those patients with atypical lesions (e.g. papules, cellulitis-like erythema) or in those patients without CKD who have classic calciphylaxis lesions (Evidence).

5.1 The skin sample should be collected by puncture, with 4 to 5 mm in diameter, preferably at the periphery of the lesion, avoiding necrotic areas. 

5.2 Biopsy is contraindicated if there is underlying infection (Evidence).

6. Treatment is based on risk factor control (discontinuing the use of warfarin, iron salts, corticosteroids, controlling alterations in mineral metabolism and intensifying dialysis), effective pain control, treatment of secondary infection (Evidence). 

7. Specific treatment of calciphylaxis should be performed with sodium thiosulfate, bisphosphonates, or hyperbaric oxygen therapy (Opinion).

## Rational

Calciphylaxis is a rare, life-threatening syndrome characterized by occlusion of microvessels in the subcutaneous adipose tissue and dermis, in addition to other tissues, resulting in extremely painful ischemic lesions^1^. However, its pathophysiology is still poorly understood. In any case, the analysis of risk factors associated with it allows us to identify possible pathophysiological mechanisms. 

Calciphylaxis is associated with the use of vitamin K inhibitors^2^. This inhibition prevents the activation of matrix Gla protein (MGP), an extracellular matrix protein synthesized in the endothelial, vascular smooth muscle and in the chondrocytes. It is a potent inhibitor of calcification^3^. Other conditions associated with vitamin K deficiency are also risk factors for the onset of calciphylaxis, such as liver disease, gastric bypass, and obesity^4^ ^,^ ^5^.

The presence of alterations in mineral metabolism, as hypercalcemia, hyperphosphatemia and PTH extremes is another risk factor. These changes might promote the appearance of all forms of vascular calcification (VC): of the intima layer of the arteries (atherosclerosis), of the media layer (arteriosclerosis, also known as Mönckeberg arteriosclerosis), valve calcification and calciphylaxis. VC is not only a passive process of mineral deposition, but rather an active one. Vascular smooth muscle cells, depending on various stimuli, modify their phenotype and express factors such as RUNX2, which is a key transcription factor for osteoblast differentiation. Once transdifferentiated, the smooth muscle cells produce matrix vesicles containing calcium and phosphorus, which will promote vessel mineralization, or calcification^6^. Although calciphylaxis frequently occurs in patients with the other forms of VC, it does not occur in 100% of patients with this complication. Similarly, the presence of the other forms of VC is not a *sine qua non* condition for the onset of calciphylaxis. ^7^. The presence of secondary hyperparathyroidism (SHPT), administration of vitamin D analogues (calcitriol or paricalcitol), hyperphosphatemia and an elevated CaxP product have often been implicated in the development of calciphylaxis. Animal models administered with high doses of PTH (hyperparathyroidism model) may develop skin necrosis similar to calciphylaxis. On the other hand, parathyroidectomy has been associated with the improvement of this complication in some patients. However, most patients with SHPT do not have calciphylaxis, and many patients with calciphylaxis do not have SHPT, suggesting the involvement of other causes. In experimental models given high doses of calcitriol, soft tissue calcifications and calciphylaxis were observed. These studies may be relevant, as calcitriol and other vitamin D analogues are often used in the treatment of SHPT. Case-control studies comparing patients with and without calciphylaxis have shown that the use of vitamin D analogues may contribute to calciphylaxis, either indirectly, through their actions to increase serum calcium and phosphorus, or directly, through their effects on vascular cells^8^. Deficiency of VC inhibitors might also act in the pathogenesis of calciphylaxis, the most studied being fetuin-A (2-Heremans-Schmid glycoprotein) and matrix Gla protein (MGP). Fetuin-A is a serum glycoprotein that binds to calcium and phosphorus forming the so-called calciproteins, thus reducing the excess of these elements in the circulation. In animal models, the presence of fetuin-A decreases organ, soft tissue and vascular calcification. Clinical studies in hemodialysis patients have shown that serum fetuin levels are lower when compared to normal subjects and have lower capacity for inhibiting calcium and phosphorus precipitation, besides correlating negatively with inflammation markers. Fetuin-A levels are reduced in patients with calciphylaxis ^9^.

Obesity is also acknowledged as a risk factor, suggesting the contribution of adipocytes in the process^10^. These cells might calcify when exposed to high phosphorus contents. Vascular endothelial growth factor (VEGF-A) is an adipokine, with potential for calcification when stimulated by bone morphogenetic protein 4 (BMP-4)^11^.

The deficiency of CD73, also referred to as NT5E (autosomal recessive disease), leads to a syndrome in which the phenotype resembles calciphylaxis. This molecule regulates the proliferation, migration and invasion of cancer cells *in vitro*. A recent study using patients from the German Registry of Calciphylaxis has assessed the genetic profile of patients with and without calciphylaxis and has demonstrated that, in addition to the CD73 gene, others, such as the vitamin D receptor and FGF-23, were associated with this complication^12^.

Other risk factors associated with calciphylaxis are: presence of CKD (in which there would be an inflammatory environment conducive to the development of calciphylaxis), female gender (with usual greater fat distribution, in addition to the possible presence of genetic factors associated with gender), diabetes mellitus (having a proinflammatory environment, malnutrition), hereditary thrombophilia, protein C deficiency, lupus anticoagulant, autoimmune diseases, repeated subcutaneous injections, accelerated weight loss, and use of other drugs, such as intravenous iron and recombinant PTH^1^.

The main differential diagnoses with chalciphylaxis are: cholesterol embolism, warfarin- or heparin-induced skin necrosis, antiphospholipid syndrome, nephrogenic systemic fibrosis, *pyoderma gangrenosum*, vasculitis, and cryoglobulinemia^13^.

The diagnosis of calciphylaxis is clinical^14^. Patients with dialysis or non-dialysis CKD presenting with complaints of severe pain (usually stabbing), skin lesions and subcutaneous induration on palpation have calciphylaxis, until proven otherwise. If subjected to skin biopsy, the main histological findings are: small vessels calcification, intimal hyperplasia and thrombosis of microvessels in adipose, subcutaneous tissue and dermis. Calcified lesions are composed of calcium and phosphorus. Inflammatory infiltrate is often observed. Arterial calcification, associated with the destruction of the endothelium and thrombosis leads to clinical manifestations of calciphylaxis. Specific stains to highlight the presence of calcium in the tissue, as the Von Kossa stain, are important for complementing the diagnosis^15^.

The treatment of calciphylaxis is multidisciplinary, involving a specialized wound care team, dermatologists, plastic surgeons, pain management specialists, among others. Nevertheless, the first item is pain control. The use of opioids may be necessary, associated with gabapentin, ketamine, as well as, spinal anesthesia, if unresponsive. As for surgical debridement, the main goals would be to remove necrotic tissue and avoid secondary infection. However, wounds are difficult to heal due to the ischemic bed of the lesions and pain during manipulation. Ulcers with signs of infection should be treated with broad-spectrum antibiotics with coverage for oxacillin-resistant, gram-negative, anaerobic *Staphylococcus aureus*, and streptococci^16^.

The main drugs used in the treatment of chalciphylaxis are:

- Sodium thiosulfate: antioxidant agent, vasodilator, which might inhibit the ability of adipocytes to induce vascular calcification. The recommended dose is 25 g, diluted in 100 mL of saline/glucose three times a week, in the last 30 to 60 minutes of hemodialysis, for approximately up to three months after healing of the lesions. The main side effects are fluid overload, hypocalcemia, prolonged QT interval, hypotension, and metabolic acidosis. However, these effects are irrelevant when administered during the last hour of dialysis. Doses for patients under conservative treatment, peritoneal dialysis, and for children are not standardized^1^ ^,^ ^17^.

- Bisphosphonates: these drugs would act by modulating the effects of alkaline phosphatase on vascular smooth muscle cells, inhibiting phosphorus transport and thus decreasing the formation of calcium phosphate crystals, besides the anti-inflammatory effects, involving macrophages, tumor necrosis factor, interleukins and other cytokines. There is no consensus on the standardized dose. One suggestion is the use of sodium pamidronate at a dose of 1 mg/kg diluted in glucose serum with a 2-hour infusion time. The dose should be repeated after 30 days^1^ ^,^ ^18^.

- Hyperbaric oxygen therapy: it is a complement justified by greater oxygen supply to damaged tissue. The exact mechanism of wound healing promoted by oxygen therapy is not fully understood. Oxygen therapy apparently improves fibroblast function, angiogenesis and neutrophil bactericidal activity, attenuating local infection^19^.

Finally, as general measures, we recommend:

1. Optimizing the dialysis dose, especially in the presence of persistent hyperphosphatemia

2. Avoiding positive calcium balance (calcium concentration in dialysate, calcium-based phosphate binders, use of vitamin D analogues).

3. Recommending parathyroidectomy in patients with secondary hyperparathyroidism and calciphylaxis.

4. Avoiding PTH levels persistently below 100 pg/mL. In this condition, the decrease in bone turnover will also decrease the buffering capacity of the skeleton, favoring the onset of hypercalcemia and/or hyperphosphatemia.
